# Solitary pulmonary nodule and ^18^F-FDG PET/CT. Part 2: accuracy, cost-effectiveness, and current recommendations*

**DOI:** 10.1590/0100-3984.2014.0087

**Published:** 2016

**Authors:** Marcos Pretto Mosmann, Marcelle Alves Borba, Francisco Pires Negromonte de Macedo, Adriano de Araujo Lima Liguori, Arthur Villarim Neto, Kenio Costa de Lima

**Affiliations:** 1 MD, MSc, Nuclear Medicine Physician at Liga Norte-Riograndense Contra o Câncer, Natal, RN, Brazil.; 2 MD, Radiologist at Liga Norte-Riograndense Contra o Câncer, Natal, RN, Brazil.; 3 PhD, Nuclear Medicine Physician at Liga Norte-Riograndense Contra o Câncer, Natal, RN, Brazil.; 4 Post Doc Fellow, Professor, Programa de Pós-Graduação em Saúde Coletiva - Universidade Federal do Rio Grande do Norte (UFRN), Natal, RN, Brazil.

**Keywords:** Solitary pulmonary nodule, Positron-emission tomography

## Abstract

A solitary pulmonary nodule is a common, often incidental, radiographic finding. The investigation and differential diagnosis of solitary pulmonary nodules remain complex, because there are overlaps between the characteristics of benign and malignant processes. There are currently many strategies for evaluating solitary pulmonary nodules. The main objective is to identify benign lesions, in order to avoid exposing patients to the risks of invasive methods, and to detect cases of lung cancer accurately, in order to avoid delaying potentially curative treatment. The focus of this study was to review the evaluation of solitary pulmonary nodules, to discuss the current role of ^18^F-fluorodeoxyglucose positron-emission tomography, addressing its accuracy and cost-effectiveness, and to detail the current recommendations for the examination in this scenario.

## Abstract (pt)

O nódulo pulmonar solitário corresponde a um achado radiológico comum, cuja detecção ocorre frequentemente de forma incidental. A investigação desta entidade permanece complexa, uma vez que existem sobreposições entre as características dos processos benignos e malignos no seu diagnóstico diferencial. Atualmente, muitas estratégias estão disponíveis para a avaliação do nódulo pulmonar solitário, e o objetivo principal consiste em caracterizar da melhor forma possível as alterações benignas, não expondo os pacientes aos riscos de métodos invasivos, e detectar corretamente os casos de câncer de pulmão, não retardando potencial tratamento curativo. O foco deste estudo é revisar a avaliação do nódulo pulmonar solitário, discutindo o papel atual da tomografia por emissão de pósitrons com 18-fluordesoxiglicose, apresentar sua acurácia e custo-efetividade, bem como salientar as recomendações atuais do exame neste cenário.

## INTRODUCTION

^18^F-fluorodeoxyglucose positron-emission tomography (^18^F-FDG PET) has been extensively evaluated in patients who present with an indeterminate solitary pulmonary nodule. In this review of the literature, we sought references on the accuracy and cost-effectiveness of the method in this scenario from electronic databases Medline and SciELO, with the terms "solitary pulmonary nodule" and "nódulo pulmonar", respectively, as well as including other articles deemed relevant. We also discuss the current recommendations for the investigation of this entity.

## ACCURACY STUDIES

Gould et al., in 2001, published a meta-analysis of ^18^FFDG PET studies in pulmonary lesions (pulmonary nodules and pulmonary masses), in which 40 studies met the inclusion criteria. Mean ages ranged from 55.5 to 70.8 years. The median prevalence of malignancy was 72.5% (interquartile range: 65-82.8%). For pulmonary lesions of any size, the sensitivity was 96.8% (95% confidence interval [CI]: 95.0-98.0%) at a point at which the receiver operating characteristic (ROC) curve corresponded to an average specificity of 77.8%. However, in pulmonary nodules, the sensitivity was 94.2% (95% CI: 89.1-97.0%) at a point at which the ROC curve showed a specificity of 83.3%. That study included a total of 450 pulmonary nodules. However, many of the selected studies had methodological limitations such as a small number of patients, lack of blinding, and possible selection biases^(1)^.

Fletcher et al. carried out a study in order to compare the accuracy of PET with computed tomography (CT) in the characterization of pulmonary nodules, although with greater methodological rigor. This prospective study included 532 patients with identified nodules in chest X-ray. Of these, 344 were evaluated in the final results by diagnostic confirmation. Sensitivities and specificities were as follows: for PET, 91.7% (95% CI: 86.6-95.0%) and 82.3% (95% CI: 75.4-87.6%), respectively; and for CT, 95.6% (95% CI: 91.3-97.9%) and 40.6% (95% CI: 33.0-48.7%), respectively. The ROC curve analysis confirmed that PET has higher accuracy compared with CT. In addition, PET correctly classified 58% of benign nodules that were incorrectly identified as malignant by CT^(2)^.

In a retrospective study of 140 patients, Grgic et al. evaluated the individual risk of malignancy from the visual interpretation of ^18^F-FDG PET images and with different cut-off points of standardized uptake value (SUVmax). The SUVmax values were higher in malignant nodules compared with benign nodules (SUVmax: 9.7 ± 5.5 vs. 2.6 ± 2.5; *p* < 0.01). More than 90% of pulmonary nodules with an SUVmax < 2.0 proved to be benign, the sensitivity, specificity, and negative predictive value (NPV) being 96.0%, 55.0%, and 92%, respectively. The greatest diagnostic accuracy was obtained with an SUVmax cut-off point of 4 (sensitivity, specificity, and accuracy of 85.0%). Visual interpretation values corresponded to 94% sensitivity, 70% specificity, and 84% accuracy. In addition to these results, SUVmax values (SUV ≥ 9.5) were predictors of lower survival^(3)^.

In order to assess the accuracy of PET in pulmonary nodules with an SUVmax < 2.5, a diagnostic challenge given the overlap of benign and malignant processes, Hashimoto et al. retrospectively analyzed 43 patients. The ^18^F-FDG uptake was graded visually (as missing, tenuous, moderate, or severe) and by the semiquantitative method. Using tenuous uptake as a cut-off point, all malignant nodules were identified-100% sensitivity, 63% specificity, 62% positive predictive value (PPV), and 100% NPV. With an SUVmax cut-off point of 1.59, there was 81% sensitivity, 85% specificity, 77% PPV, and 89% NPV^(4)^.

A number of factors influence the spatial resolution of PET equipment, which is currently 5-7 mm^(5)^. The contrast of the lesion decreases in processes with a diameter less than twice the spatial resolution of the system used^(5)^. Herder et al. addressed this issue in a retrospective study including 36 pulmonary nodules with a diameter ≤ 1 cm. The authors reported 93% sensitivity, 77% specificity, 72% PPV, and 94% NPV. It is important to point out that only 8 of the 36 nodules had a diameter ≤ 0.8 cm, suggesting that further studies in this line of research are needed in order to confirm the impact of PET in this scenario^(5)^.

In addition to the dimensions of the solitary pulmonary nodule, another variable that should be considered when evaluating the diagnostic performance of PET is the degree of metabolic behavior of different tumors, because false-negative results can be obtained. Yap et al. demonstrated that PET presented a sensitivity of only 33% for patients with pure bronchioloalveolar carcinoma without an invasive component. ^(6)^

Other papers have presented results for different subgroups of patients. Evangelista et al. evaluated the use of PET/CT in the investigation of pulmonary nodules in 29 patients diagnosed with breast cancer. For nodules ≥ 0.8 cm, the method presented 77% sensitivity, 85% specificity, 85% PPV, 69% NPV, and 80% accuracy^(7)^. Cistaro et al. investigated 18 pediatric patients with bone sarcomas^(7)^. The visual analysis of PET/CT images showed 90.3% sensitivity, 87.5% specificity, 87.5% PPV, 90.3% NPV and 88.9% accuracy^(8)^. Kagna et al.^(9)^ studied patients defined as high risk for lung cancer regarding the ^18^F-FDG PET/CT investigation of a pulmonary nodule. They obtained the following results for the visual analysis: 94% sensitivity, 70% specificity, 66% PPV, 95% NPV and 80% accuracy.

One technique that has been evaluated in pulmonary nodules is obtaining late complementary images, with comparison of SUVs in the first stage (approximately 60 minutes after injection of ^18^F-FDG) and in a second stage (approximately 120 minutes after the injection). Matthies et al. evaluated 36 patients with that modality. The SUVs of malignant nodules were 3.66 ± 1.95 and 4.43 ± 2.43 in the first and second scans, respectively, compared with 1.14 ± 0.64 and 1.11 ± 0.7, respectively, for benign nodules. There was a significant increase of 20.5 ± 8.1% in malignant nodules (*p* < 0.01). With an SUV cut-off point of 2.5 in the first scan, the authors obtained 80% sensitivity and 94% specificity. Adding the criteria of a 10% increase in the SUV between the two acquisitions increased sensitivity to 100% and decreased specificity to 89%^(10)^.

These results were not confirmed in subsequent studies conducted in areas endemic for tuberculosis. Chen et al. included 31 pulmonary nodules with an SUV < 2.5 to evaluate the accuracy of PET in two phases. An increase of more than 10% in the SUV was observed in 60% of the benign nodules and in 62% of malignant nodules, resulting in sensitivity of 62%, specificity of 40%, and accuracy of only 52%^(11)^. A study involving a population in South Africa evaluated 30 patients with ^18^F-FDG PET/CT in two phases and obtained results suggesting that this method does not allow a malignant pulmonary nodule to be distinguished from tuberculoma. The median (interquartile range) SUVmax values for malignant lesions, tuberculomas, and other benign conditions were 6.7 (3.2-13.1), 7.6 (5.9-12.7) and 1.4 (0.9-1.8), respectively, those for tuberculomas being significantly greater than those for the other benign etiologies (*p* < 0.05). The median (interquartile range) variations in SUVmax between the two phases were 19.5% (12.9%-41.5%), 13% (5.9%-22.7%) and -11.3% (-25.3%-10.1%) for malignant lesions, tuberculomas and other benign processes, respectively, with no significant difference between tuberculosis and malignancy. When the authors used an SUVmax = 2.5 as the cut-off point, the sensitivity and specificity were only 85.7% and 25%, respectively; when they excluded tuberculomas, those values were 85.7% and 100%, respectively^(12)^, closer to other data in the international literature. Kim et al. also addressed this issue, including 25 patients with tuberculoma evaluated with ^18^F-FDG PET/CT in two phases. A diagnosis of active pulmonary tuberculoma was defined as that confirmed by positive culture in sputum or bronchoalveolar lavage fluid samples or by polymerase chain reaction. However, there was no diagnostic confirmation in the inactive tuberculoma group, and other diseases presenting as pulmonary nodules might therefore have been included. In comparison with the patients in the inactive tuberculoma group, those in the active tuberculoma group showed a significantly higher mean SUVmax (2.3 ± 0.75 vs. 0.79 ± 0.15), as well as a significantly greater mean variation in the SUVmax (8.07% ± 7.77% vs. -3.83 ± 6.59)^(13)^. More recently, a metaanalysis including 8 studies showed that the accuracy of ^18^FFDG PET/CT was similar between the standard protocol and the two-phase protocol, although the latter was found to have slightly higher specificity^(14)^.

In a prospective study conducted in Rio de Janeiro, Martins et al. included 32 patients and obtained results that differed from those of other studies performed in areas endemic for tuberculosis. The authors reported a 40.6% prevalence of malignancy and, using an SUVmax cut-off point of 2.5, obtained 92.9% sensitivity, 72.2% specificity, 72.2% PPV, 92.9% NPV, and 81.2% accuracy^(15)^.

Tsushima et al. evaluated the accuracy of ^18^F-FDG PET/CT in solitary pulmonary nodules with non-solid component in 53 patients in Japan. With an SUVmax cut-off point of 1.5, the diagnostic performance was 100% sensitivity, 96.4% specificity, 96.2% PPV, 100% NPV, and 100% accuracy^(16)^.

In a retrospective study conducted in Korea, the authors evaluated 100 pulmonary nodules with 40% prevalence of malignancy, in order to compare PET/CT, CT alone, and PET alone, in terms of the diagnostic parameters. Three radiologists independently evaluated pulmonary nodules with those three modalities. The sensitivity of CT alone, PET alone, and PET/CT was 82%, 88% and 88%, respectively, whereas the specificity was 66%, 71%, and 77%, respectively; the accuracy was 72%, 78%, and 81%, respectively; the PPV was 61%, 67%, and 72%, respectively; and the NPV was 84%, 90%, and 90%, respectively. Therefore, PET/CT showed better specificity than did PET alone or CT alone (*p* < 0.05). The mean SUVmax in malignant and benign processes was 8.2 ± 4.5 and 3.4 ± 2.9, respectively, and the difference was significant (*p* < 0.001)^(17)^. Kim et al. published a study with similar method but involving a population in the United States. A total of 42 patients were evaluated retrospectively. The respective values for CT, PET, and PET/CT were as follows: 93%, 69%, and 97% for sensitivity; 31%, 85%, and 85% for specificity; 75%, 91%, and 93% for PPV; 67%, 55%, and 92% for NPV; and 74%, 74%, and 93% for accuracy. In summary, the combination of anatomical and metabolic imaging resulted in a significant improvement in the accuracy of the characterization of pulmonary nodules^(18)^.

Using a qualitative method to evaluate the accuracy of PET/CT in pulmonary nodules in 56 patients, an Israeli group obtained the following results: 96% sensitivity, 83% specificity, 84% PPV, 96% NPV, and 89% accuracy^(19)^. A retrospective study involving 209 patients in Turkey also assessed the accuracy of ^18^F-FDG PET/CT in the differential diagnosis of benign and malignant disease. In that study, an SUVmax of 4 provided the best distinction between diseases (84.0% sensitivity, 70.0% specificity, 81.8% PPV, 73.0% NPV, and 78.4% accuracy)^(20)^.

Patient breathing during the acquisition of PET images is a factor that can create artifacts, usually representing increased volume of the lesion or a reduction in its uptake^(21)^. Werner et al. addressed this issue in a study of 18 patients, evaluating the size and uptake of the lesions using the standard protocol and a protocol including respiratory synchronization. The authors demonstrated that with the incorporation of the proposed technique, there was a 15.5% reduction in the area of the lesion (*p* = 0.014), a 10.3% reduction in its axial dimension (*p* = 0.007), and a 44.5% reduction in its volume (*p* = 0.025), as well as a 22.4% increase in the SUVmax (*p* < 0.001). Despite these results, further studies are needed in order to determine whether the use of this method will change the accuracy of the test, compensating for the costs of and operational difficulties related to respiratory synchronization^(21)^.

Another imaging modality that deserves mention is dynamic contrast CT. In a prospective study, Swensen et al.^(22)^ evaluated 550 indeterminate pulmonary nodules. Of those, 356 met the inclusion criteria and had appropriate follow-up for diagnostic definition. Using a delayed enhancement threshold of 15 Hounsfield units (HU), the authors obtained the following diagnostic values: 98% sensitivity, 58% specificity, 77% accuracy, 68% PPV, and 96% NPV, an increase ≤ 15 HU therefore proving to be strong predictor of benignity. It is of note that although that was a multicenter study, it discussed the generalization of results obtained for areas endemic for tuberculosis, given the small number of patients included in the study. In addition, the method has some limitations, such as the fact that it cannot be used in persons who are allergic to the contrast agent or in those with renal failure, as well as technical injection problems and registration failure due to subject breathing. Christensen et al.^(23)^ compared dynamic CT with delayed enhancement and ^18^F-FDG PET, in terms of the evaluation of solitary pulmonary nodules. A total of 42 nodules were included, with a malignancy rate of 60%. For dynamic CT, sensitivity, specificity, PPV, and NPV were 100%, 29%, 68%, and 100%, compared with 96%, 76%, 86% and 93%, respectively, for PET. Another study^(24)^ compared the diagnostic accuracy of dynamic CT with ^18^F-FDG PET/CT. Using a ≥ 25 HU increase as the threshold for suggesting malignancy, the authors found that the sensitivity, specificity, accuracy, PPV, and NPV were 81%, 93%, 85%, 96%, and 71%, respectively, for dynamic CT and 96%, 88%, 93%, 94%, and 92%, respectively, for PET/CT.

Magnetic resonance imaging (MRI) has also been evaluated for investigation of pulmonary nodules. In a prospective study involving 40 patients, Stolzmann et al. compared the detection rate, location, and size of pulmonary nodules using three modalities: low dose CT, PET, and MRI^(25)^. Detection rates were similar (*p* > 0.05): CT revealed 66 nodules in 34 patients (85%); and MRI detected 58 nodules in 33 patients (83%). Furthermore, the nodules detected were significantly smaller in MRI. With the development of hybrid PET/MRI equipment, it might be possible to investigate nodules with this method. However, studies that are more robust are needed in order to compare PET/CT and PET/MRI in this scenario.

## COST-EFFECTIVENESS STUDIES

Health care systems, most of which have limited financial resources, face the daunting task of having to decide where to allocate their resources, often at the expense of spending in other areas. Cost-effectiveness studies have been widely accepted and used in order to answer questions of this nature with the best available evidence^(26)^.

Gambhir et al.^(27)^ created a decision analysis model comparing different strategies: wait and watch, in which all patients were observed with serial X-rays or chest CT scans to determine whether the nodule showed malignant growth rate, thus selecting patients for biopsy or surgery; surgery, in which all patients were subjected to thoracotomy to remove the nodule, if resectable; chest CT, in which patients were subjected to high-resolution CT before the decision was made to perform biopsy or surgery; and CT and PET, in which both imaging modalities were performed before the decision was made to perform biopsy or surgery. The base case for the initial analysis was a 64-year-old white male who was a smoker (1.5 pack/day), with a 2.5 cm nodule and life expectancy of 14.8 years (pretest probability of 0.83). Costs were included in accordance with Medicare reimbursement rates, and effectiveness was determined in accordance with the average life expectancy. To compare each strategy with the least invasive strategy (wait and watch), the incremental cost-effectiveness ratio (ICER) was calculated. Analyzing ICERs calculated with a threshold of US$50,000 as the acceptable cost per year of life saved, the following results were obtained: the wait and watch strategy was the one with best cost-effectiveness ratio in patients with low pretest probability of malignancy (≤ 0.12); in patients with intermediate probability of malignancy (0.12-0.69), the best strategy was CT and PET; for patients in which the probability was between 0.69 and 0.90, the strategy with chest CT proved superior; for those in which the probability was above 0.90, the most cost-effective strategy was surgery. In a sensitivity analysis in which the PET diagnostic parameters were reduced by 15%, the CT and PET strategy remained cost-effective in a range of pretest probabilities between 0.17 and 0.45.

Dietlein et al.^(28)^ published a model from the perspective of the German health care system and used a decision tree that differed from that proposed by Gambhir et al.^(27)^. In that analysis, the initial evaluation of a solitary pulmonary nodule already includes X-ray and chest CT. The strategies evaluated were as follows: wait and watch, with serial CT scans at 3, 6, 12, 18, and 24 months; biopsy, assuming CT-guided transthoracic biopsy; surgery; and PET. Another peculiar feature of the study conducted by Dietlein et al.^(28)^ was to include in the PET strategy not only the diagnostic values of pulmonary nodule but also decisions based on mediastinal staging. The costs were obtained in accordance with the reimbursement from the national health care system, whereas, for effectiveness, a search of the literature was performed for secondary data in accordance with life expectancy depending on the diagnosis and comorbidities. For the comparison between strategies, the ICER was calculated. Applying a default threshold of EUR50,000 per year of life saved and varying the probability of nodule malignancy, the following results were obtained. Compared with the wait and watch strategy, the PET strategy showed a better ICER in a range of probabilities between 0.10 and 0.70. In patients with a 0.5 probability, the wait and watch strategy proved to be the most cost-effective. In patients with a high probability of cancer (between 0.75 and 0.95), the surgical strategy showed the best ICER.

In 2003, Gould et al.^(29)^ presented a decision model with significant differences in comparison with previous ones. The study evaluated 40 possible combinations of 5 diagnostic interventions: CT, FDG-PET, biopsy, surgery, and watchful waiting. The target population included adult patients with a noncalcified nodule on chest X-ray. For the first time, a Markov model was used in order to estimate the long-term outcomes as well as costs for patients with pulmonary malignant and benign nodules. The study was conducted from the perspective of society in US population. In addition, the values of effectiveness used became quality-adjusted life-years (QALYs). The main results presented by the authors were as follows: 1) effectiveness and cost-effectiveness of the different management strategies of the solitary pulmonary nodule depend on the malignancy pretest probability and, to a lesser extent, on the risk of surgical complications; 2) chest CT is recommended as the initial test in almost all circumstances except when the pretest probability is very high; 3) the nonselective use of FDG PET is highly effective in facilitating the diagnosis of pulmonary nodules, although this modality has proven to be more cost-effective when the pretest values are discordant from those of the chest CT, that is, at intermediate post-test probabilities; 4) the aggressive use of biopsy and surgery is highly effective and cost-effective, after obtaining image results. Sensitivity analysis showed that different post-test probabilities (after chest CT) change the strategy, with a better cost-effectiveness ratio. At very low post-test probabilities (< 2%), the watchful waiting strategy was the most appropriate; at probabilities between 2% and 20%, the biopsy strategy was the most appropriate; at probabilities between 20% and 69%, the FDG PET strategy was the most appropriate; and for post-test probabilities above 70%, the surgical strategy showed the best cost-effectiveness ratio. Surgery was also the preferred method, without inclusion of other modalities, when the pre-test probability was above 90%. In addition, in patients with high surgical risk, the FDG PET strategy presented a cost below US$100,000 per QALY gained at post-test probabilities between 35% and 84%.

Lejeune et al.^(30)^ developed a decision analysis model to compare the cost-effectiveness of FDG PET with that of other modalities in the management of solitary pulmonary nodules from the perspective of the French health care system. Three alternatives were evaluated: wait and watch, PET and CT+PET. The base case for analysis was defined as a 65-year-old male smoker (1.5 pack/day), with a noncalcified 2 cm pulmonary nodule (43% risk of malignancy). Comparisons, based on the ICER, were made between different strategies and the wait and watch strategy. The CT+PET strategy showed a lower cost-effectiveness ratio, with a value of EUR3,022 per life-year gained (LYG). Sensitivity analysis showed that for patients with a low probability of malignancy (between 0.3% and 5%), the wait and watch strategy was best. In those with probabilities between 5.7% and 87%, the most appropriate strategy was CT+PET, with an ICER between EUR1,159 and EUR48,350 per LYG.

Comber et al.^(31)^ evaluated the impact of including contrast-enhanced CT scans in the management of pulmonary nodules. From the perspective of the Australian health care system, the authors created four diagnostic strategies evaluated by a decision analysis model: 1) conventional CT; 2) conventional CT followed by dynamic CT; 3) conventional CT followed by PET; and 4) conventional CT followed by dynamic CT and PET. For the comparison between strategies, the cost per patient was calculated. In addition, effectiveness was compared using an intermediate outcome measure called management accuracy, in which the mean value of this outcome corresponds to the proportion of patients appropriately managed. Thus, the measure of cost-effectiveness was presented as the incremental cost-accuracy ratio. The results showed that in the base case analysis (i.e., at a 54% prevalence of malignancy), the strategy with the lowest cost-effectiveness ratio was dynamic CT followed by PET (US$ 12,059.18 per patient). Sensitivity analysis confirmed that this strategy remains the most cost-effective at malignancy probability values below 54%. At a prevalence of over 60%, the use of dynamic CT appears to be the superior strategy, although the cost-effectiveness of conventional CT is comparable to that of dynamic CT when the prevalence of disease is extremely high (> 90%). Other studies, using decision analysis models developed in Italy and Australia^(32,33)^, have also obtained results similar to those previously described.

## CURRENT RECOMMENDATIONS FOR ^18^ F-FDG PET/CT IN THE INVESTIGATION OF SOLITARY PULMONARY NODULES

Many international guidelines provide recommendations for the management of solitary pulmonary nodules. Table 1 shows the current recommendations of the American College of Chest Physicians^(34)^.

**Table 1:** Summary of recommendations for the management of patients with indeterminate solitary pulmonary nodules.

Patient with a solid indeterminate solitary pulmonary nodule with a diameter > 0.8 cm
• A functional image, preferably PET, is suggested for nodule characterization, in an individual with low to moderate pre-test probability (5% to 65%). • PET can be indicated with the aim of pre-treatment staging and not for characterizing the nodule in a patient with high pre-test probability (> 65%). • Follow-up with CT (3 to 6 months, 9 to 12 months and 18 to 24 months, using low dose technique without contrast ) is suggested in the following circumstances: – when the clinical probability of malignancy is very low (< 5%); - when the clinical probability is low (< 30% to 40%) and the results of the functional image tests are negative, resulting in a very low post-test probability of malignancy; – when the biopsy is inconclusive and the injury is not hypermetabolic on PET; – when the patient prefers a nonsurgical approach. • Biopsy and/or surgical resection is suggested in individuals with evidence of malignant growth in serial images (unless there are specific contraindications). • Biopsy is suggested in the following circumstances: – when the pre-test clinical probability and imaging findings are discordant; – when the probability of malignancy is low to moderate (10% a 60%); – when there is suspicion of a benign lesion that requires specific medical treatment; – when the patient desires proof of a malignant diagnosis prior to surgery, especially if the risk of surgical complications is high . • Surgery is suggested in the following circumstances: – when the clinical probability of malignancy is high (> 65%); – when the nodule is intensely hypermetabolic on PET or positive on another functional image test; – when the biopsy is suggestive of malignancy; – when the patient prefers to undergo a definitive diagnostic procedure.
Patient with a solid indeterminate solitary pulmonary nodule with a diameter ≤ 0.8 cm and no risk factors for lung cancer
• Nodules ≤ 0.4 cm do not need to be monitored, but the patient must be informed of the potential risks and benefits. • Nodules measuring > 0.4 cm and ≤ 0.6 cm should be reevaluated after 12 months without the need for follow-up if they remain unchanged. • Nodules > 0.6 cm and ≤ 0.8 cm should be reevaluated after 6 to 12 months and again after 18 to 24 months if they remain unchanged.
Patient with a solid indeterminate solitary pulmonary nodule with a diameter ≤ 0.8 cm and one or more risk factors for lung cancer
• Nodules ≤ 0.4 cm should be reevaluated after 12 months, without the need for follow-up if they remain unchanged. • Nodules measuring > 0.4 cm and ≤ 0.6 cm should be reevaluated after 6 to 12 months and again after 18 to 24 months if they remain unchanged. • Nodules > 0.6 cm and ≤ 0.8 cm should be reevaluated after 3 to 6 months, after 9 to 12 months and again after 24 months if they remain unchanged.
Patient with a non-solid (ground-glass) indeterminate pulmonary nodule
• For nodules ≤ 0.5 cm, monitoring is not mandatory. • Nodules > 0.5 cm should be monitored annually for at least three years.
Patient with a part-solid (> 50% ground-glass) indeterminate pulmonary nodule
• For nodules ≤ 0.8 cm, it is suggested that the patient be reevaluated after approximately 3, 12, and 24 months, and that annual CT scans be obtained for an additional 1 to 3 years. • For nodules > 0.8 cm, it is suggested that the chest CT scan be repeated after 3 months, and that that be followed by evaluation by PET, biopsy, or surgical resection for any remaining nodules. • Nodules > 1.5 cm should immediately be submitted to evaluation by PET, biopsy, or surgical resection.

Adapted from American College of Chest Physicians^(34)^.

**Figure 1:**
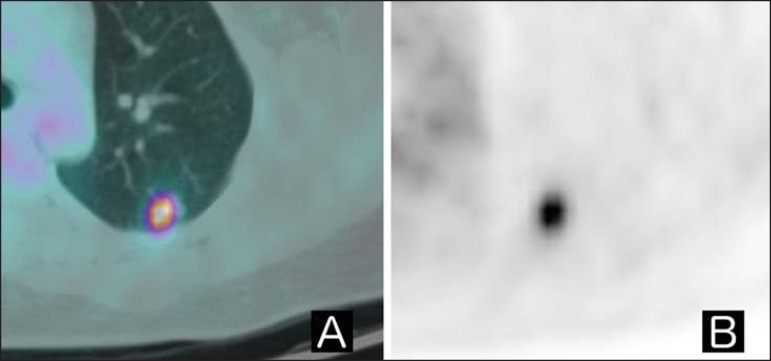
**A:** Axial fused ^18^F-FDG PET/CT image. **B:** Axial PET image. Patient with history of epidermoid cervical carcinoma, referred for indeterminate pulmonary nodule research. Upon ^18^F-FDG PET/CT examination, the nodule displayed increased glycolytic metabolism (SUVmax = 5.6). She was submitted to surgery, which confirmed the hypothesis of involvement of the underlying disease.

**Figure 2:**
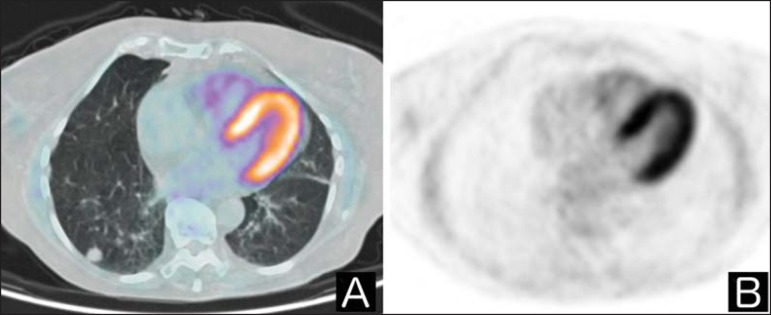
**A:** Axial fused ^18^F-FDG PET/CT image. ^B:^ Axial PET image. Patient with no history of cancer referred for investigation of an indeterminate pulmonary nodule. Upon ^18^F-FDG PET/CT examination, the pulmonary nodule showed no anomalous accumulation of the tracer.

**Figure 3:**
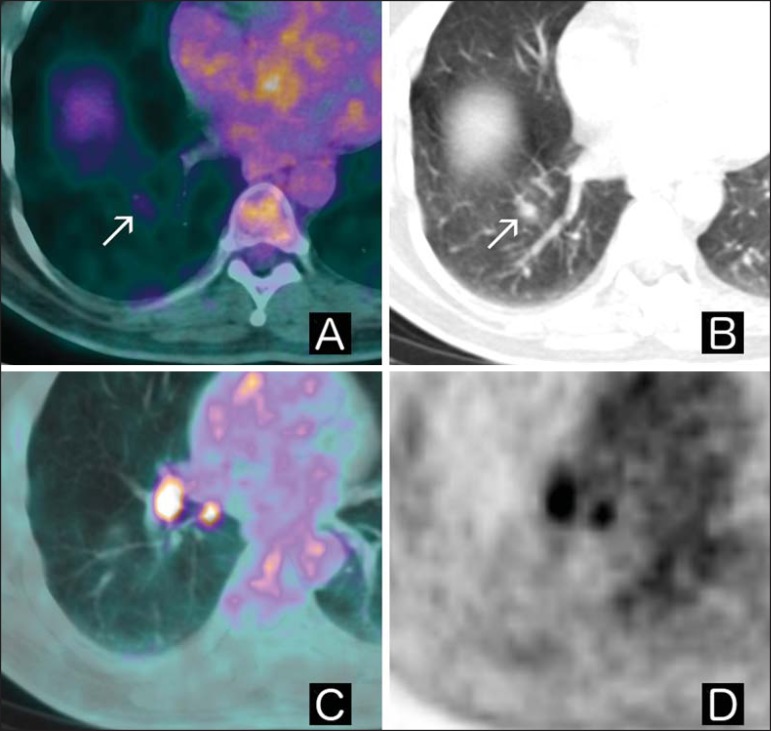
**A:** Axial fused ^18^F-FDG PET/CT image. **B:** Axial chest CT image. **C:** Axial fused ^18^F-FDG PET/CT image. **D:** PET axial image. Patient with a history of gastrointestinal stromal tumor presenting with indeterminate pulmonary nodule in the right lower lobe in a previous CT scan. Upon ^18^F-FDG PET/CT examination, the nodule displayed slightly increased glycolytic metabolism when compared with normal lung parenchyma (arrows in **A** and **B**), together with hypermetabolic ipsilateral hilar lymph nodes (**C** and **D**). The patient underwent surgery, which further confirmed the diagnosis of tuberculosis.

The ^18^F-FDG PET technique is still not on the Brazilian Unified Health Care System list of approved procedures for the investigation of solitary pulmonary nodules, although its future incorporation is being discussed. On the basis of evidence in the literature, the Brazilian Oncology Society and the Brazilian Society of Biology, Nuclear Medicine, and Molecular Imaging published a list of recommendations for the use of PET/CT test with ^18^FDG in oncology^(35)^. The authors of that list broadly asserted that PET is indicated for the evaluation of solitary pulmonary nodules ≥ 1.0 cm (class IA; i.e., with adequate clinical evidence in the medical literature). Comparisons between PET and ^18^F-FDG PET/CT are shown in Figures 1 and 2. Although there have been many studies demonstrating the potential benefits of incorporating this modality in developed countries, it should be emphasized that the generalization of the results of cost-effectiveness studies to other health care systems can be problematic, particularly in countries with areas endemic for infectious granulomatous diseases (Figure 3) and with greater restrictions on health care investment. Given these considerations, we believe that cost-effectiveness studies conducted in the context of the Brazilian health care system can provide important additional information to facilitate the decision-making process related to whether or not this new technique should be incorporated, as well as to help identify the subgroup of patients in which this modality can be best employed.

In April 2014, the Brazilian National Ministry of Health published three ordinances that incorporate PET/CT into the public health care system. The recommendation was made by the National Commission for the Incorporation of New Technologies and covers the use of PET/CT for the following purposes: 1) the clinical staging of potentially resectable non-small cell lung cancer^(36)^; 2) the detection of exclusively hepatic, potentially resectable metastasis of colorectal cancer^(37)^; and 3) the staging and evaluation of the response to treatment of Hodgkin and non-Hodgkin lymphoma^(38)^.

## CONCLUSION

Currently, an indeterminate solitary pulmonary nodule is a common finding, and many strategies are available to manage such nodules. The development of noninvasive methods with greater accuracy and lower costs could help better characterize these nodules and thus make better use of health resources, as well as allowing the choice of modalities to be individualized, which could provide greater benefits and reduce exposure to potential risks. Using ^18^F-FDG PET/CT in the evaluation of solitary pulmonary nodules seems to be most appropriate in patients with an intermediate probability of malignancy. However, cost-effectiveness studies aimed at determining the budgetary impact at the national level can provide information that will help health care professionals make the best use of this technique.
